# Courtyard geometry’s effect on energy consumption of AlKharga city residential buildings, Egypt

**DOI:** 10.1038/s41598-024-60487-8

**Published:** 2024-05-15

**Authors:** Ahmed M. Seddik Hassan, Reham Fathy Ahmed Abd El Aal, Asmaa Abd elmoneim Fahmi, Sherif Mohamed Ahmed Ali, Mohamed I. M. Abdelhady

**Affiliations:** 1 Department of Architectural Construction Technology, Faculty of Technology and Education, Beni-Suef University, Beni Suef, Egypt; 2 Department of Civil and Architectural Constructions, Faculty of Technology and Education, Suez University, P.O. Box: 43221, Suez, Egypt

**Keywords:** Courtyard, Courtyard placement, Residential buildings, Hot dry areas, Energy simulation inside the building, Energy science and technology, Engineering

## Abstract

The research aim is to clarify the effect of courtyard placement, the ratio between length and width, and courtyard orientation on energy consumption in residential buildings in hot and dry climates, to seek planning and designing alternatives for new cities and new residential complexes that are consistent with the environment and climate and save the consumption of energy used in the buildings. The research method was conducted through Design Builder software for simulation purposes. The reference model with the 157.25 m^2^ which accommodates a central square courtyard measuring 5 m × 5 m, on a residential building model in the New Valley Governorate of Kharga City, Egypt. The courtyard simulation is aimed to determine Less energy consumption inside the different case studies, in 9 courtyard placements The studied alternatives for Courtyard Placement, are (the center of the building, sub facades, and main facades). The different ratios are (1:1, 1.25:1, 1.5:1–1.75:1, 2:1, 2.25:1, 2.5–1). The longitudinal axis of the Courtyard has been oriented to the east–west direction for all placements, and north–south direction for all placements. Also, (orientation angle is Zero), it offered the percentages of better and worst cases in each position of the courtyard. The research findings suggest that the best Placement of the Courtyard that achieved the highest rate of saving of energy consumed inside the used building model was at the southwestern façade, with a saving rate of 18.73%. Then, the Placement of the Courtyard at the northwestern and southeastern facades with a saving rate of 17.91%, with a length-to-width ratio (2.5:1) if the longitudinal axis of the Courtyard is oriented in the north–south direction, Through the study, we conclude that the placement and orientation of the courtyard and its regular formation have contributed to rationalizing energy consumption in residential buildings, the study reached some important standards that could represent a methodological framework for designing contemporary residential buildings with an energy-efficient inner courtyard.

## Introduction

The problem of the research is that the designs of most buildings in hot dry areas disregard the impact of the climate conditions surrounding such buildings on the climate formation inside buildings. Moreover, new architectural elements have been introduced, that are not consistent with such conditions and don’t take them into account, such as the great development of modern construction styles resulting in the development of the architectural structure, freedom in facades design, the spread of the vertical construction style in most urbanization areas in the desert. This has resulted in a similarity of architectural designs in all countries despite the difference in climate conditions.

One of the most architectural elements characterizing the Arab heritage urban fabric is the inner Courtyard. Arabs and Muslims used it in their architecture due to its consistency with the environmental needs and requirements and the cultural and social concepts. Therefore, the inner Courtyard is considered one of the most important architectural solutions contributing to the confrontation of climate problems, particularly in hot dry areas. It also has great importance in the environmental design system of residential buildings, particularly in the provision of natural lighting and ventilation of high quality, resulting in energy efficiency^1^.

Many recent studies recommended reviving the style of buildings with courtyards in modern architecture, particularly in hot areas, as they are more appropriate for such climates and more energy-efficient than the modern styles of modern residential buildings^2^. The studies that addressed the inner Courtyard as a climatic mechanism proved that it is a climatic mechanism appropriate to the hot and dry regions, i.e., in the regions between latitudes 15°-30°, north or south of the equator, where most Arab countries, which adopted the inner courtyards in their traditional architecture, are located in these regions. In a study conducted to determine the best shape of the buildings in various climate regions, it was found that the buildings with courtyards are the most appropriate for the regions of hot dry climates, constituting the most significant percentage of the Arab Countries^3^.

According to the space characteristics of the Courtyard, its Placement has an important value for architecture and urbanism and is an influential factor in the extent of functional, environmental, and formational efficiency of a residence. The urban fabric in the hot and hot dry areas in the Arab countries reflects styles, in which the courtyard placement represents an important dimension in the formation, function, and environment of urbanism and distinguishing its identity. The study will address the effect of the inner courtyard placements on the consumption of energy in residential buildings in hot dry areas.

This study attempted to investigate the various parameters of the courtyard, such as placement, orientation, and elongation of the courtyard and their effects on the energy consumption inside architectural spaces as a tool for Energy saving. One of the Kharga city in the New Valley Governorate, Egypt residential models is chosen for testing and simulation as a case study of the present study.

### Research limits

The application of the study will be limited to residential buildings in hot, dry areas. An example of these areas is the Arab Republic of Egypt, New Valley Governorate—Kharga City.

### Research limitations

- Lack of research studies in the field of study, as most of the studies that dealt with the courtyard were related to ventilation and temperature and their effect on thermal comfort within architectural spaces.
- The inability to obtain electricity bills and meter readings after changing the electrical meters to prepaid meters, to calibrate the case study and compare it with the results of the program.

## Inner courtyard

The inner courtyard is one of the most important architectural and symbolic features of the residential buildings in heritage architecture. It is also deemed as the heritage architectural system in treating the hot environment. It is also one of the most important principles of environmental, sustainable, and green architecture in Islamic housing architecture due to its ability to adapt to various climate conditions and meet various environmental, aesthetic, social, health, and psychological requirements^4^.

### Importance of the courtyard in designing modern residential buildings at hot dry areas

**Figure 1:**
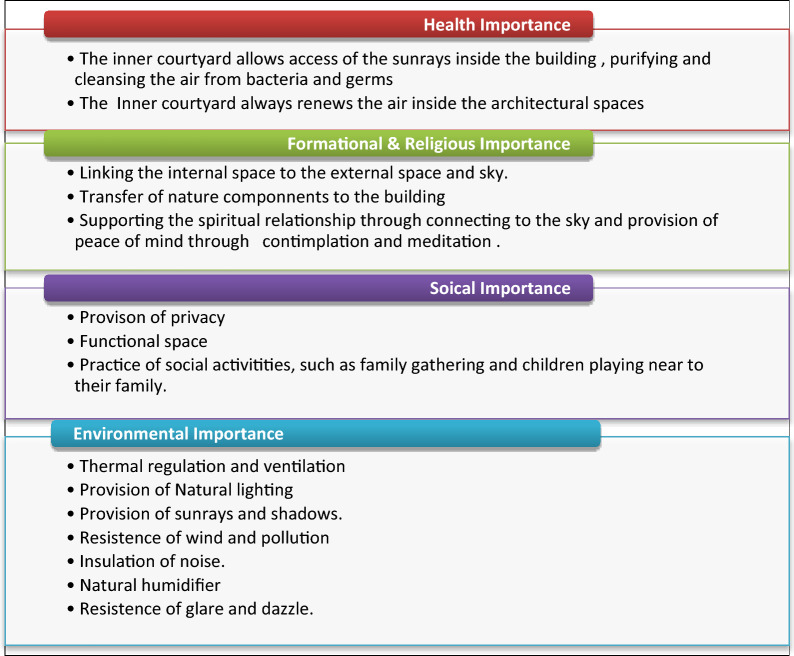
Importance of incorporation of inner courtyard in residential buildings.

Many researchers concluded that: “The adjoining buildings with inner courtyards are the most appropriate to the hot dry climate”^5^. The studies emphasized that the buildings with inner courtyards are the most buildings forming shadows during the sun movement during the day, and in the case of buildings of multiple floors, the shadows increase^6^. The courtyards reduce areas of roofs and walls exposed to the sunrays and other climate factors to a large extent and achieve the greatest amount of shadows, consequently protecting the building from the intense impact of the sunrays in hot areas^7^, as the inner Courtyard acts as a thermal regulator inside the residence through the provision of thermal comfort by the provision of cold shaded air substituting the hot air inside rooms surrounding it. The Courtyard is significantly important in various environmental^3^, formational, religious^4^, health, and social^8^ aspects, as shown in Fig. 1.

### Inner courtyard definition

There are various linguistic, terminological, and architectural definitions of the inner Courtyard, including the following:

#### Linguistic and terminological definition of the inner courtyard

The Courtyard is linguistically defined as a space in the middle of a building mass or an area extended in front of or around it. For the terminological definition of the Courtyard, it is an architectural means providing privacy and protection or a closed space structure composed of continuous or semi-continuous walls and overlooked by spaces of direct beneficial relevance^9^.

#### Architectural definition of the courtyard

The inner Courtyard is a space open to the sky and surrounded by building masses. It may be located inside or outside the building. Most windows of the rooms overlook it. It is used as an architectural component controlling the climate in the building design, as it moderates temperature and illuminates and ventilates the internal spaces^10^. It may also be defined as a part of the surrounding general space, which is surrounded by the building’s internal spaces to adapt to the environmental, formational, and social conditions^9^.

### Placement of the courtyard and residence climate

The studies proved that the Courtyard reduces the temperature during the day inside buildings by varying values based on several factors, including air movement and coldness, amount of shadows, used building materials and their colors, and their degree of sunray dispersal^11^. The design of the residence around the Courtyard is one of the best methods to address environmental problems of the climate, particularly in the desert areas, as the inner Courtyard acts as a regulator of the temperature inside the residence, night and day. It provides ventilation and protects residences against hot wind. These are achieved through orienting such Courtyard and, adjusting its Placement for such a residence, and shaping its walls and building materials. The Courtyard also helps to achieve communication of the user with the external space. Moreover, the inner Courtyard allows the possibility to cultivate plants and trees and set up fountains inside it, resulting in improving and moderating the climate condition using such components^7^. Consequently, the Courtyard contributes to raising the efficiency of internal spaces surrounding it, reducing the energy consumption inside the residence.

### Placement of courtyard and thermal cycle system

The Placement and formation of the Courtyard are the most important factors influencing the quality and efficiency of the thermal cycle system of the Courtyard, which is related to the day, noon, and night, as the areas of its walls exposed to the sun and shading amounts resulting from the orientation and Placement significantly help in adjusting the movement of the cold and hot air and the daily frequent thermal cycle of the Courtyard. The good Placement of the Courtyard adds environmental value to the residence spaces. The Courtyard helps provide natural lighting and ventilation, reduces energy consumption, and depends on the use of natural energy sources^12^.

**Figure 2:**
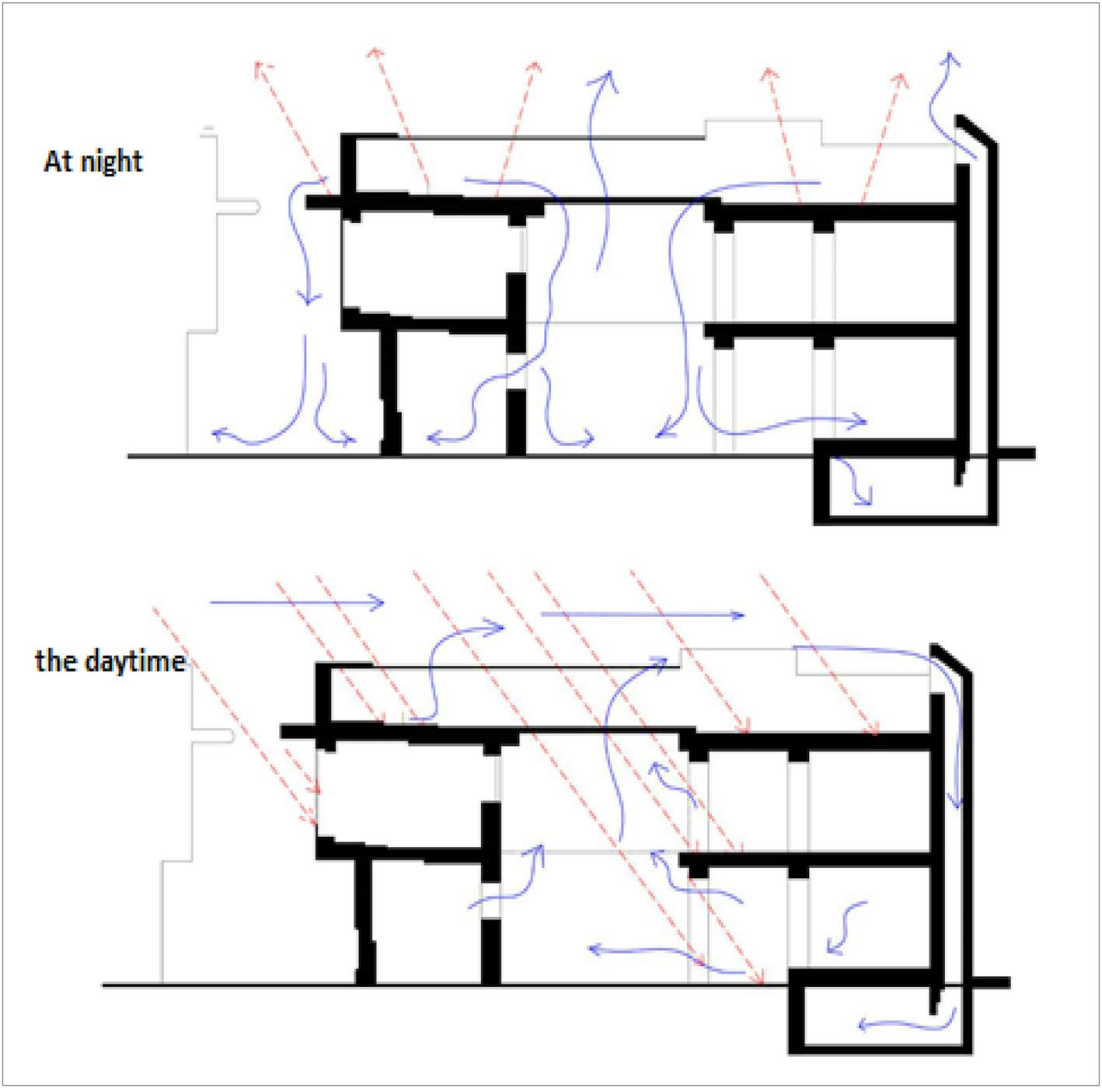
Thermal performance of the inner courtyard at night and during the daytime (Continuous lines in blue represent the air movement, while dashed lines in red color represent the solar and thermal radiation)^15^.

The Courtyard acts as a thermal regulator in the residential buildings, as it cools the internal spaces. In its performance of its function, the Courtyard acts as a store of the cold air at night. The idea of the Courtyard's internal work depends on the phenomenon of significant variation of temperature between night and day, as at night, the Courtyard reradiates the amounts of the sunrays stored in its ground and walls all day to the sky; meanwhile, the cold air is stored therein to make use of the courtyard coldness during the next day^13^. During the daytime, the performance of the Courtyard differs when it is exposed to the sun's rays, as the weight of hot air decreases and rises upward and the cold air is drawn through the windows of rooms to substitute the hot air^14^. In addition, shading a vast area of its ground, reducing the reflected sunrays, and using trees, fountains, and climbing plants that insulate walls help cool the temperature of the Courtyard, and the moderately cold air gathers in layers and flows inside the spaces overlooking the Courtyard^15^. See Fig. 2.

### Shape of the courtyard and its relation to residence spaces

**Figure 3:**
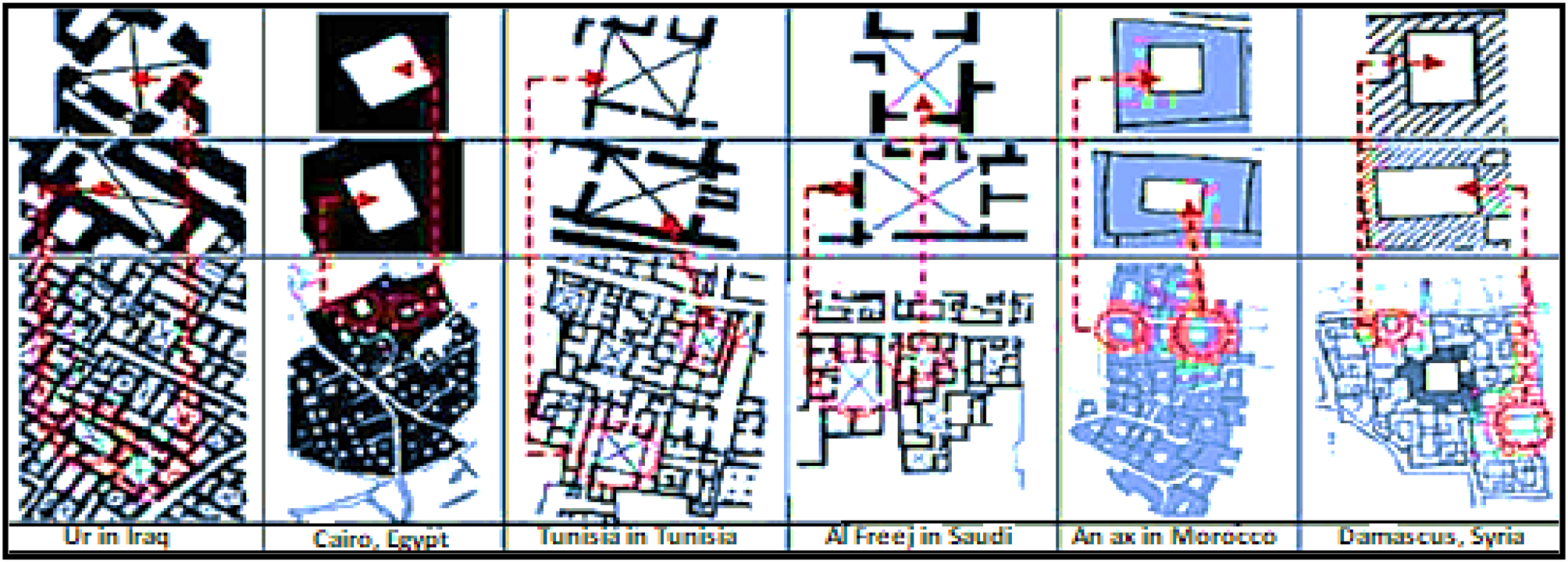
The prevailing formation of the courtyard in heritage buildings that diversifies between square and rectangular shapes^11^.

The courtyard shape and Placement are important dimensions in the engineering and organization of the internal spaces. It’s common knowledge in heritage architecture that the shape of inner courtyards takes a purely geometric shape, as the vast majority of them take on two basic shapes, the square and rectangular shapes^8^ as shown in Fig. 3. The courtyard shape is also made up of the distribution or grouping of the architectural spaces around it and the nature of the space relations between the inner Courtyard and the spaces overlooking it. Other factors influencing the shape and form of the Courtyard derived from the heritage and traditional architecture often include the land area, building size, total rooms, spaces overlooking the Courtyard, and the functional purpose for which the Courtyard was set up. All these factors are mainly related to the social aspects of the owner of the residence, such as the number of family members, social status, and financial status.

This formation contributed to the symmetry of surrounding spaces. Moreover, the regularity of the geometric shape of the Courtyard between the square and rectangular shapes and adjusting its Placement to be consistent with the cardinal directions or in a clear relation with the cardinal directions works on attaining the orientation of the residence spaces^11^.

### Inner courtyards orientation

**Figure 4:**
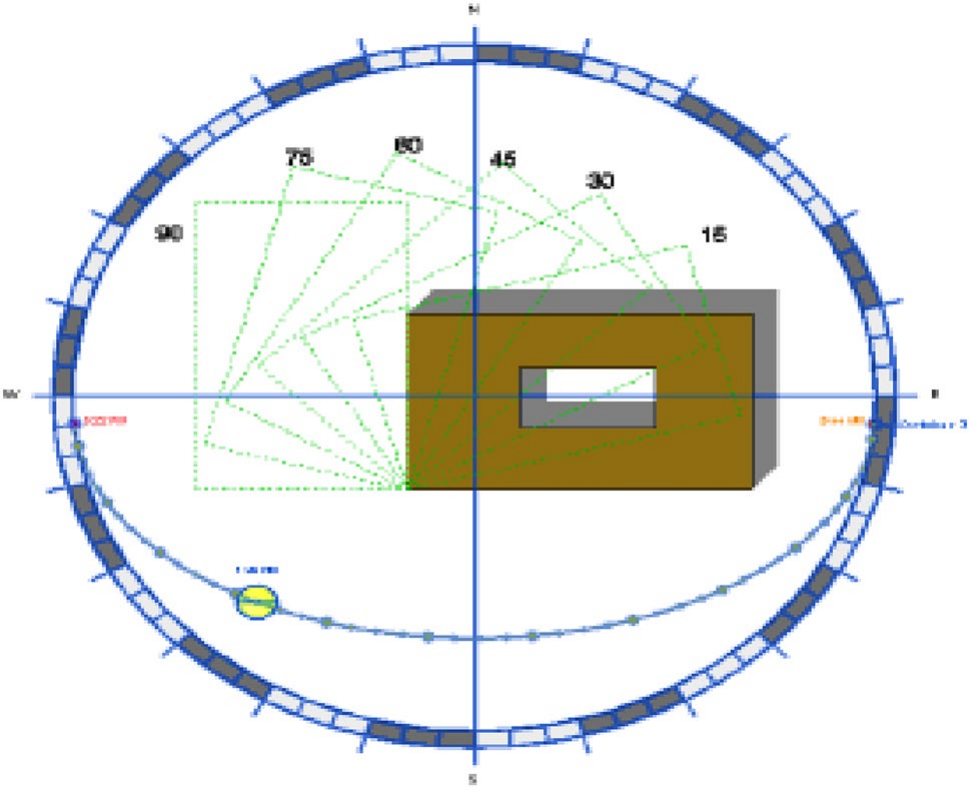
Varying the orientation of the courtyard from 0° to 90°^2^.

Following the general orientation of the Courtyard in terms of the cardinal directions, the most important element of the climatic system of the Courtyard, and through the proper orientation of the Courtyard, we can attain the minimum exposure to the sun rays in the summer and the maximum exposure to the sun rays in the winter, and we can as well take advantage of the wind movement in the courtyard ventilation. The proper orientation of the Courtyard helps achieve thermal comfort inside the building^13^. Figure 4 shows varying the orientation of the Courtyard from 0° to 90°.

### Courtyard geometric measurements

**Figure 5:**
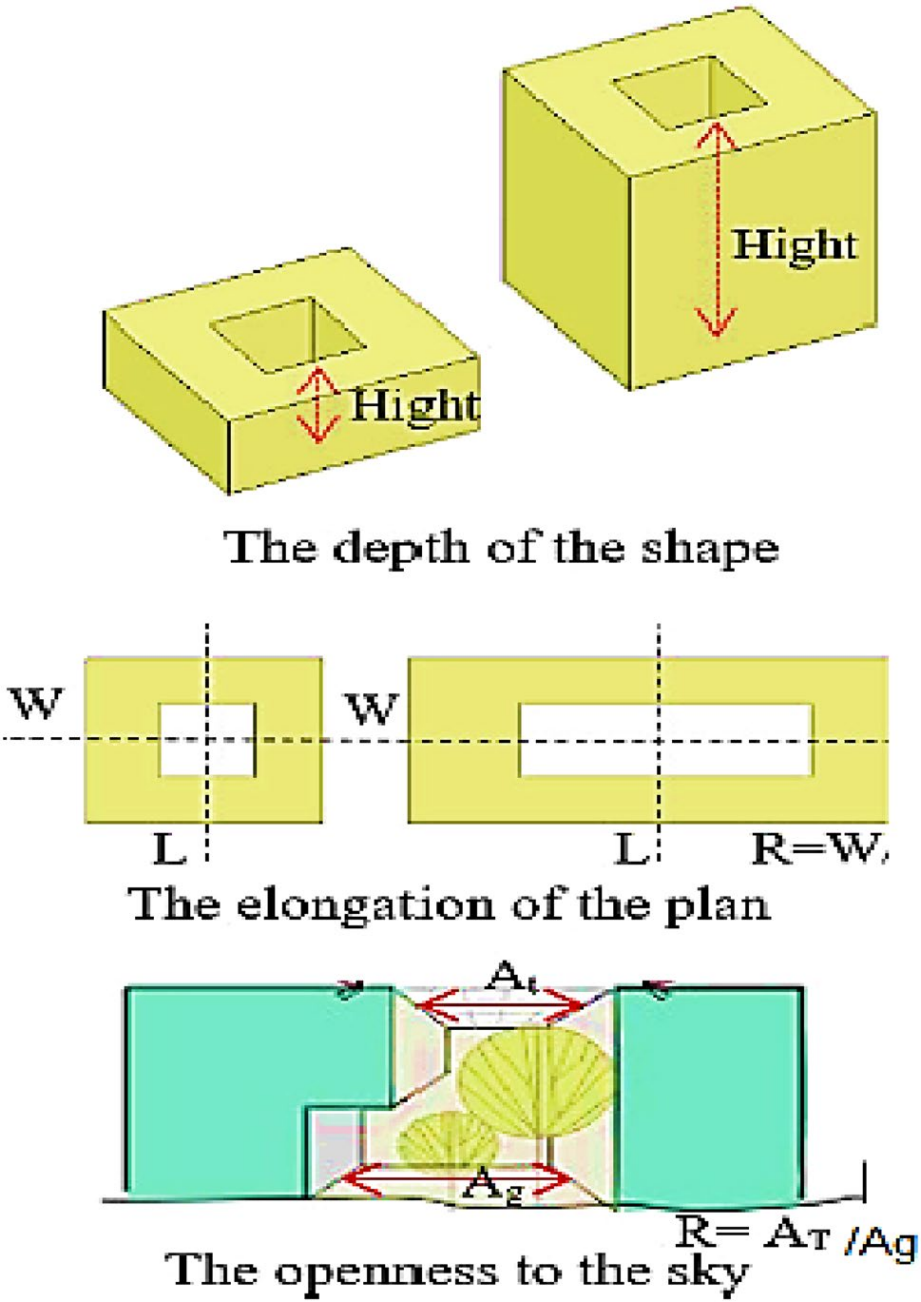
The geometric dimensions of courtyard^2^.

The geometric dimensions of the Courtyard influence the performance of such Courtyard. The studies demonstrated that the effectiveness of any courtyard and its performance efficiency as a climatic mechanism is based on many factors, the most important of which is the courtyard dimensions, including length, width, and height. The height of the courtyard walls is the most important factor affecting the access of solar radiation to the Courtyard. For instance, increasing the height of courtyard walls from one floor to two floors would delay the access to the sun’s rays by about two hours or more. Therefore, the courtyard walls and their height must be appropriate to their dimensions in the horizontal plane. Therefore, the researchers recommend that the courtyard dimensions in the horizontal plan shouldn’t exceed the height of its walls. The geometric dimensions of the Courtyard could be identified in three variables, including^2^ as shown in Fig. 5.

- Courtyard Depth: The ratio between the courtyard perimeter and height. Such Courtyard will be deemed as deep if this ratio is less than 3.
- Courtyard Elongation: The ratio between the courtyard width and length.
- Openness to the Sky: the ratio between the courtyard land area and courtyard upper area, which equals 1 in case of lack of prominences in the Courtyard upper area.

### Placement of the courtyard and its relation to residence

**Figure 6:**
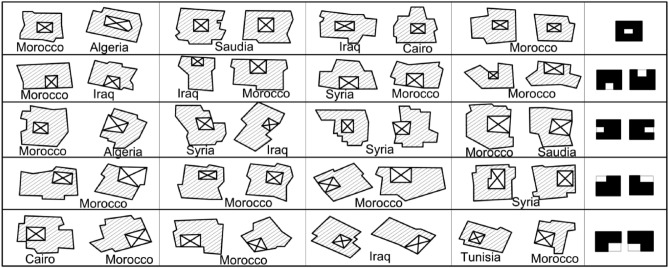
Placement of the courtyard in some different models on the Arab Heritage Urbanism^11^.

The heritage residential urban fabric in Egypt and the Arab region includes several similar instances of courtyard placement. It has been noted from the residences spreading in the Arab region that the Courtyard located in the middle of the residence spaces is the most common, followed by the Courtyard surrounded by residence spaces on three sides. Then, the corner courtyard or the Courtyard surrounded by residence spaces on two sides is less common. Then, the Placement of the Courtyard surrounded by residence spaces on one side is rare^11^. Therefore, the Placement of the Courtyard has been selected to be in the center of the building as the main Alternative for the study. Figure 6 shows selected models of heritage buildings in Arab countries illustrating the Placement of the Courtyard in the residence in the heritage architecture.

The placements of the Courtyard can be described as follows.

- Courtyard placement in the center of the building.
- Courtyard placement in the middle of the main facades (Northern, eastern, southern, western).
- Courtyard placements on sub-facades (northeastern, northwestern, southeastern, southwestern), show Table 1 the different placements of Courtyard in residential buildings.

**Table 1:** The different placements of courtyards in residential buildings.

Case no.	Courtyard placement in the building	
1	Center of the building	
2	The Placement is in the middle of the northern façade	
3	The Placement is in the middle of the eastern façade	
4	The Placement is in the middle of the southern façade	
5	The Placement is in the middle of the western façade	
6	The Placement on the northeastern façade	
7	The Placement on the north-western façade	
8	The Placement on the south-eastern façade	
9	The Placement on the south-western façade	

## Method and materials

The second part of the study addresses the potential of reducing energy consumption by studying the relationship between the residence and the Placement of the inner Courtyard, the research methodology included conducting an experiment to determine the impact of the courtyard's various design parameters on energy consumption, Using Kharga city in the New Valley Governorate, Egypt as a case study, the experiment included simulating energy consumption different courtyard cases, The research method was conducted through Design- Builder software for simulation purposes.

### The study area

The building selected for the simulation is located in Kharga city in the New Valley Governorate, Egypt. Kharga city is located in the Egyptian Western Desert, specifically in the northern part of Kharga Depression. The city is 72 m above sea level. It’s bordered to the north by Gabal Altayr and to the northwestern by Gabal Tarwan. The city is located in the middle of urban centers in the Kharga Depression^17^.

**Figure 7:**
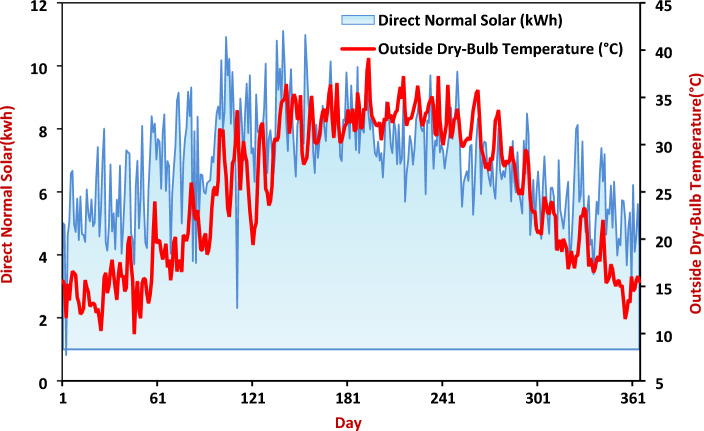
Daily average temperature and levels of solar radiation in Kharga City.

The study area is astronomically located between latitudes 25°, 27° north and longitudes 30°, 32° east^18^. The area is known for its harsh climate conditions, especially during the summer, so, it is a prominent model of the hot dry desert region, requiring special architectural treatments that are consistent with the environment and climate and save the consumption of energy used in residential buildings. Figure 7 shows the average temperature and solar radiation in Kharga city.

#### Imran city of Kharga

The city has a distinctive traditional architectural style that was created by the conditions of the surrounding environment, the needs and social conditions, and the customs and traditions of the people of the city, As for the modern residential neighborhoods in the city of Kharga, they were formed after the implementation of the local government system in the New Valley Governorate in 1986 AD, and the city witnessed a major urban expansion after the preparation and approval of the general plan for the city from 1986 AD until now, most of the urban expansions are located in the western direction, and accordingly, Kharga was divided into two urban areas^7^:

- **Old Kharga**, is dominated by a compact urban fabric and contains many valuable heritage buildings, and the urban style of the Old Kharga neighborhood is compatible with the hot desert nature.
- **New Kharga and the new extension areas**, the residential site consists of several residential blocks that are repeated on the site, leaving spaces between them, noting that these blocks and sites are repeated in most Egyptian cities despite the different environmental and climatic conditions of these cities.

### Methodology

#### Evaluation of the building's climate performance

At this stage, a database is prepared for the building, which includes determining the climatic region in which the building is located, determining its location and area, the type of building (residential building), and preparing a climatic data file for the city.

#### Determine designing alternatives for the courtyard

##### Placement of courtyard

**Figure 8:**
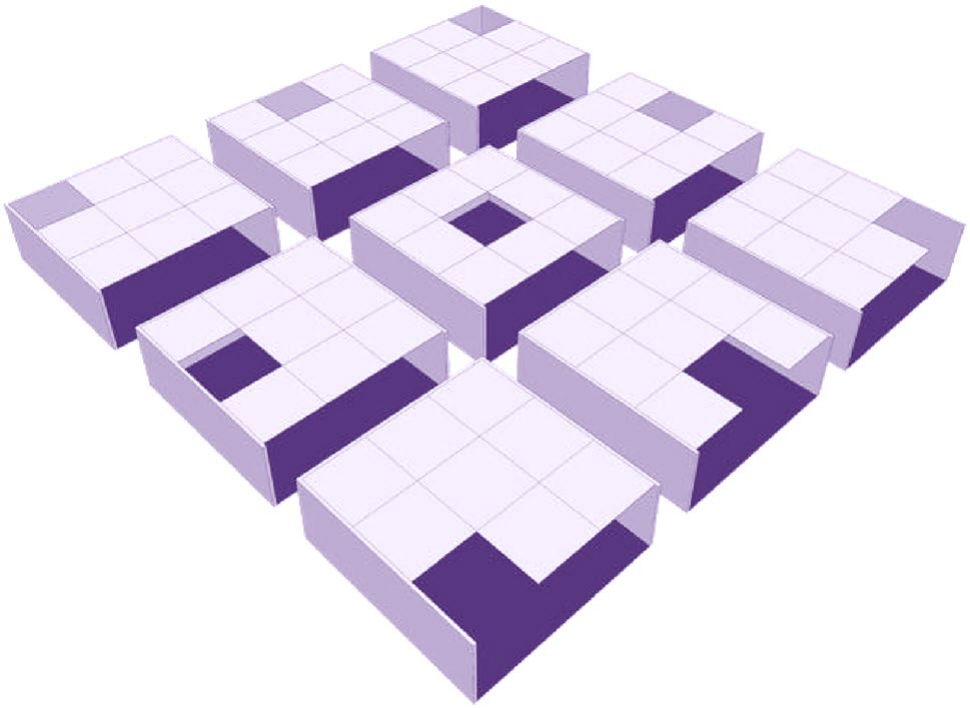
The 9 courtyard placements.

Many design alternatives have been developed for the Placement of the Courtyard at the residence, including (the center of the building, northern façade, western façade, southern façade, eastern façade, northwestern façade, northeastern façade, southwestern façade, southeastern façade), Fig. 8 shows the 9 Courtyard Placement, the main Alternative will be compared to is the square Courtyard at the center of the building, Table 1 shows the different placements of Courtyard in residential buildings.

##### Courtyard elongation

The Courtyard has been designed in various placements by the following ratios (1:1, 1.25:1, 1.5:1–1.75:1, 2:1, 2.25:1, 2.5–1).

##### Courtyards orientation

**Figure 9:**
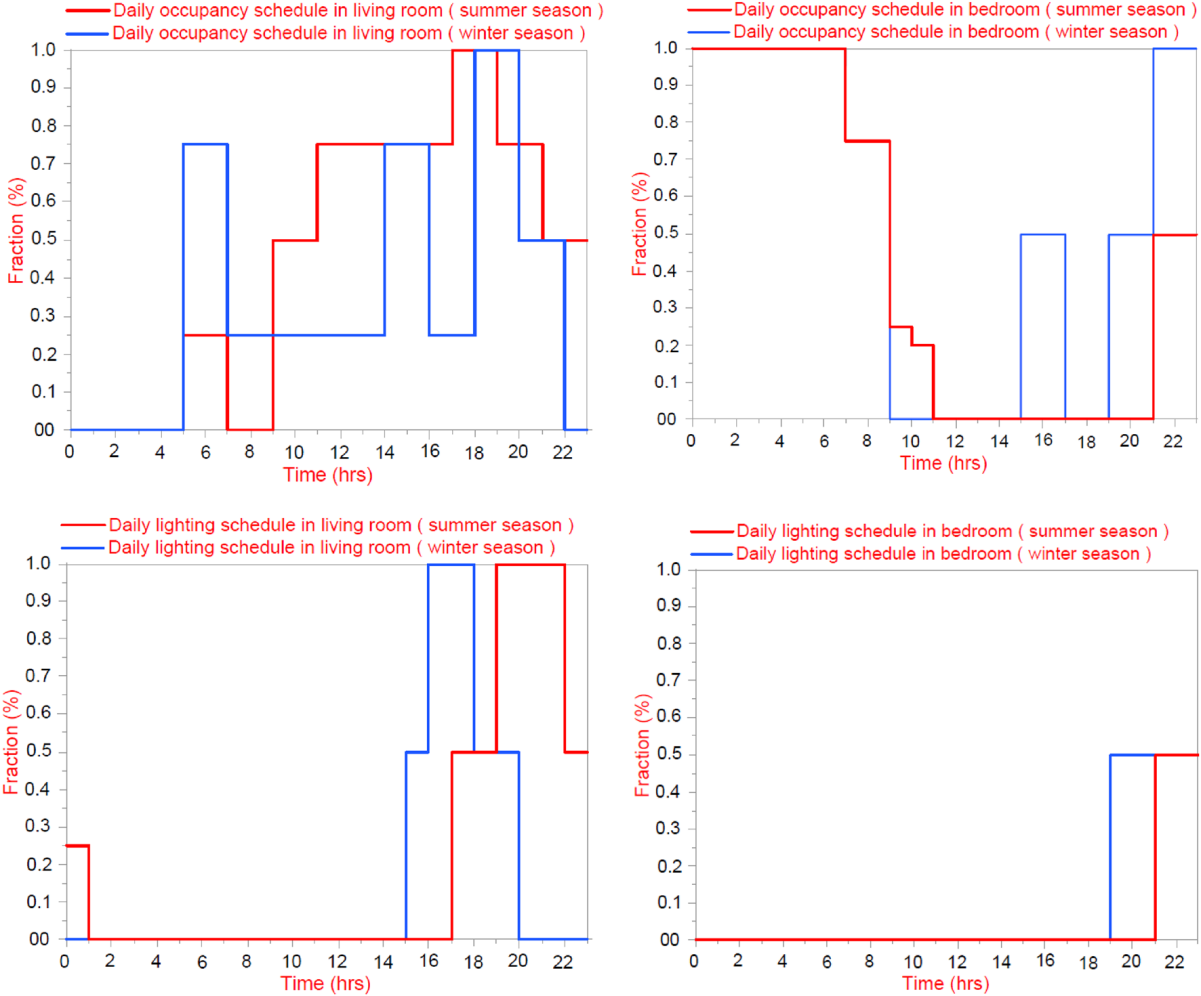
Daily occupancy schedule for different apartment rooms.

The longitudinal axis of the Courtyard has been applied at the east–west direction for all placements and ratios. In addition, the longitudinal axis of the Courtyard has been changed to be at north–south direction for all placements and ratios (the orientation angle is Zero) (Fig. 9).

#### Simulation

##### Reference model for simulation

**Figure 10:**
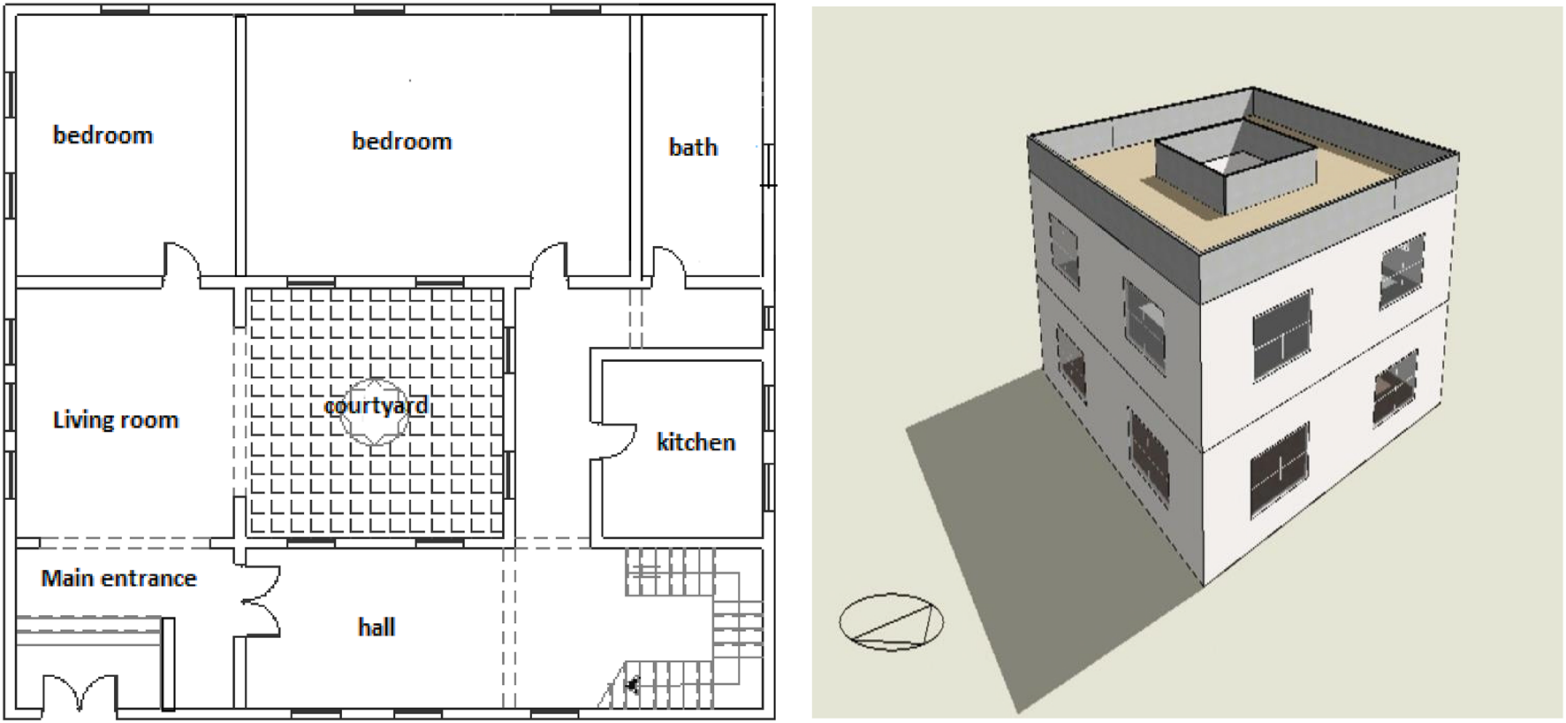
The study building model.

The building subject of the study is a residential building of two floors, the total area of which is 157.25 Sq m, with a square-shaped inner Courtyard in the middle of the building spaces measuring 5 m × 5 m, and is a residence of one family, as shown in Fig. 10. DesignBuilder software has been used to design the building. Table 2 shows the different specifications of the building, which have been used as inputs in the simulation process, such as the materials used in the construction, the Weather Data File, and different energy loads.

**Table 2:** Simulation Specifications.

Phases	Name	Description/total area	Notes/value (if any)
Residential building description	Type of building	Residential	
	Number of floors	2 (Ground floor + first floor)	
	Total Area	157.25 m^2^	
	Area Courtyard	25 m^2^	
	Gross wall area (Building only)	522.72 m^2^	
	Gross wall area courtyard only	193.6 m^2^	
	Gross wall area (Building and Courtyard)	716.32 m^2^	
	Building height	10.9 m	
	Dimensions of the Courtyard	5 × 5 m	
Program inputs	Type of brick used	Solid baby bricks, thickness 12 cm	U-Value = 2.703 w/m^2^-k
	Types of Window Glass	Sig Clr glass 6 mm	SHGC = 0.810 LT = 0.881 U-Value = 6.121 w/m^2^-k
	Window-To-Wall Ratio (WWR)	20%	
	Flat roof	Without thermal insulation	U-Value 2.677 w/m^2^
	Drawing	Using drawing program window—DesignBuilder	
	Building orientation	0.0°	
	conditioned- with refrigeration only	Type: Split no fresh air Setting the Air-conditioners at a temperature of 24°	
	Artificial Lighting	2.5 W/m^3^ (LED)	
	Number of occupants	7	
	Weather Data File	Kharga city, Egypt	
	(HVAC) Daily occupancy schedule	Variant as shown in Fig. 9	

With regard to the occupancy schedule for the building rooms used in the simulation, the results of the survey conducted by Attia^19^ in Egyptian cities are based on the average surveyed air conditioning and lighting schedule in different spaces for several apartments in some Egyptian cities, which is consistent to the case study^16^.

##### Energy simulation

The investigation of placement, elongation, and orientation of the courtyard was carried out using DesignBuilder software, one of the most reliable and widely used tools in several researches related to energy, has been used. DesignBuilder is dynamic simulation software used to calculate a wide range of thermal performance data, in addition to the energy consumption of a virtual building model with the EnergyPlus engine. In essence, EnergyPlus™ brings together the best capabilities and features of BLAST and DOE-2^16^.

DesignBuilder software has been used to assess the effect of the inner Courtyard on energy consumption in modern residential buildings by utilizing the inner Courtyard at different placements derived from Arab heritage urbanism see Fig. 6 to conclude the best Placement resulting in the highest rate of saving of energy consumption. The Placement of the Courtyard has been selected to be in the center of the building as the main Alternative of the study, to which the other alternatives will be compared due to the widespread of such courtyard placement in residential buildings.

A different model of the building is formed by the adjustment in placement, elongation, and orientation of the courtyard which includes the different cases of building, The nine main placements of reference model for various simulation sets, are shown in Table 1, the Courtyard has been designed in various placements by the following ratios (1:1, 1.25:1, 1.5:1- 1.75:1, 2:1, 2.25:1, 2.5–1) and the simulations of the courtyard orientation the longitudinal axis of the Courtyard has been applied at the east–west direction for all placements and ratios. In addition, the longitudinal axis of the Courtyard has been changed to be a north–south direction for all placements and ratios (orientation angle is Zero), to determine the best length-to-width ratio for the Courtyard and the best Placement and orientation, achieving the thermal comfort and the highest rate of saving of energy consumption inside the residence, Table 3 shows energy consumption values for the study cases, of the reference building.

**Table 3:** Energy consumption values of the cases study.

Courtyard placement	orientation of the longitudinal axis of the Courtyard	length to width proportion	CO2 emissions (kg CO2)	energy consumption (kwh)	Proportion of energy savings comparing to the (main Alternative) %
Center of the Building	East–West	**1:1**	**17,896**	**28,338**	**Main alternative 0%**
		1:1.25	17,904	28,351	− 0.05%
		1:1.5	17,926	28,388	− 0.18%
		1:1.75	17,951	28,430	− 0.32%
		1:2	17,980	28,479	− 0.50%
		1:2.25	18,013	28,533	− 0.68%
		1:2.5	18,041	28,579	− 0.84%
	North–South	1:1	17,896	28,338	0%
		**1:1.25**	**17,889**	**28,326**	**0.04%**
		1:1.5	17,903	28,349	− 0.04%
		1:1.75	17,917	28,373	− 0.12%
		1:2	17,939	28,411	− 0.26%
		1:2.25	17,958	28,442	− 0.37%
		1:2.5	17,978	28,475	− 0.48%
Northern position	East–West	1:1	16,872	26,634	6.01%
		1:1.25	16,753	26,435	6.72%
		1:1.5	16,680	26,314	7.14%
		1:1.75	16,608	26,195	7.56%
		1:2	16,547	26,094	7.92%
		1:2.25	16,496	26,009	8.22%
		**1:2.5**	**16,451**	**25,934**	**8.48%**
	North–South	**1:1**	**16,872**	**26,634**	**6.01%**
		1:1.25	16,985	26,822	5.35%
		1:1.5	17,077	26,975	4.81%
		1:1.75	17,158	27,110	4.33%
		1:2	17,230	27,231	3.91%
		1:2.25	17,293	27,335	3.54%
		1:2.5	17,351	27,431	3.20%
Eastern position	East–West	**1:1**	**16,224**	**25,564**	**9.79%**
		1:1.25	16,393	25,845	8.80%
		1:1.5	16,533	26,076	7.98%
		1:1.75	16,652	26,275	7.28%
		1:2	16,757	26,450	6.66%
		1:2.25	16,855	26,613	6.09%
		1:2.5	16,940	26,753	5.60%
	North–South	1:1	16,224	25,564	9.79%
		1:1.25	16,046	25,269	10.83%
		1:1.5	15,921	25,061	11.56%
		1:1.75	15,804	24,868	12.25%
		1:2	15,697	24,692	12.87%
		1:2.25	15,607	24,541	13.40%
		**1:2.5**	**15,522**	**24,400**	**13.90%**
Southern position	East–West	1:1	16,682	26,320	7.12%
		1:1.25	16,547	26,095	7.92%
		1:1.5	16,455	25,943	8.45%
		1:1.75	16,367	25,797	8.97%
		1:2	16,287	25,665	9.43%
		1:2.25	16,217	25,548	9.85%
		**1:2.5**	**16,149**	**25,436**	**10.24%**
	North–South	**1:1**	**16,682**	**26,320**	**7.12%**
		1:1.25	16,807	26,528	7.34%
		1:1.5	16,908	26,696	5.80%
		1:1.75	16,995	26,840	5.29%
		1:2	17,071	26,968	4.83%
		1:2.25	17,139	27,081	4.44%
		1:2.5	17,200	27,183	4.10%
Western position	East–West	**1:1**	**16,125**	**25,401**	**10.36%**
		1:1.25	16,303	25,697	9.32%
		1:1.5	16,451	25,941	8.46%
		1:1.75	16,574	26,147	7.73%
		1:2	16,683	26,328	7.10%
		1:2.25	16,785	26,497	6.50%
		1:2.5	16,871	26,640	6.00%
	North–South	1:1	16,125	25,401	10.36%
		1:1.25	15,934	25,085	11.48%
		1:1.5	15,797	24,857	12.28%
		1:1.75	15,669	24,645	13.03%
		1:2	15,552	24,453	13.71%
		1:2.25	15,451	24,283	14.31%
		**1:2.5**	**15,356**	**24,128**	**14.86%**
Northeastern position	East–West	**1:1**	**15,328**	**24,072**	**15.05%**
		1:1.25	15,376	24,152	14.77%
		1:1.5	15,410	24,208	14.57%
		1:1.75	15,432	24,244	14.45%
		1:2	15,445	24,267	14.37%
		1:2.25	15,460	24,290	14.28%
		1:2.5	15,442	24,261	14.39%
	North–South	1:1	15,328	24,072	15.05%
		1:1.25	15,270	23,977	15.39%
		1:1.5	15,214	23,885	15.71%
		1:1.75	15,160	23,796	16.03%
		1:2	15,103	23,701	16.36%
		1:2.25	15,034	23,587	16.77%
		**1:2.5**	**14,995**	**23,523**	**17.00%**
Northwestern position	East–West	**1:1**	**15,239**	**23,926**	**15.57%**
		1:1.25	15,298	24,023	15.23%
		1:1.5	15,339	24,091	14.99%
		1:1.75	15,367	24,137	14.82%
		1:2	15,385	24,167	14.72%
		1:2.25	15,385	24,166	14.72%
		1:2.5	15,385	24,166	14.72%
	North–South	1:1	15,239	23,926	15.57%
		1:1.25	15,169	23,810	15.98%
		1:1.5	15,102	23,699	16.37%
		1:1.75	15,035	23,590	16.75%
		1:2	14,967	23,478	17.15%
		1:2.25	14,906	23,376	17.51%
		**1:2.5**	**14,837**	**23,263**	**17.91%**
Southeastern position	East–West	**1:1**	**15,150**	**23,779**	**16.09%**
		1:1.25	15,182	23,831	15.90%
		1:1.5	15,198	23,858	15.81%
		1:1.75	15,203	23,867	15.78%
		1:2	15,200	23,861	15.80%
		1:2.25	15,173	23,817	15.95%
		1:2.5	15,157	23,790	16.05%
	North–South	1:1	15,150	23,779	16.09%
		1:1.25	15,103	23,702	16.36%
		1:1.5	15,056	23,624	16.63%
		1:1.75	15,007	23,543	16.92%
		1:2	14,954	23,456	17.23%
		1:2.25	14,906	23,376	17.51%
		**1:2.5**	**14,837**	**23,263**	**17.91%**
Southwestern position	East–West	**1:1**	**15,064**	**23,637**	**16.57%**
		1:1.25	15,106	23,707	16.34%
		1:1.5	15,129	23,744	16.21%
		1:1.75	15,138	23,759	16.16%
		1:2	15,137	23,757	16.17%
		1:2.25	15,134	23,752	16.18%
		1:2.5	15,092	23,683	16.43%
	North–South	1:1.25	15,064	23,637	16.57%
		1:1.5	15,007	23,543	16.92%
		1:1.75	14,949	23,447	17.26%
		1:2	14,889	23,349	17.61%
		1:2.25	14,828	23,248	17.96%
		1:2.5	14,774	23,158	18.28%
		**1:1.25**	**14,696**	**23,029**	**18.73%**

Bold values that give the best energy.

#### Results and discussion

**Figure 11:**
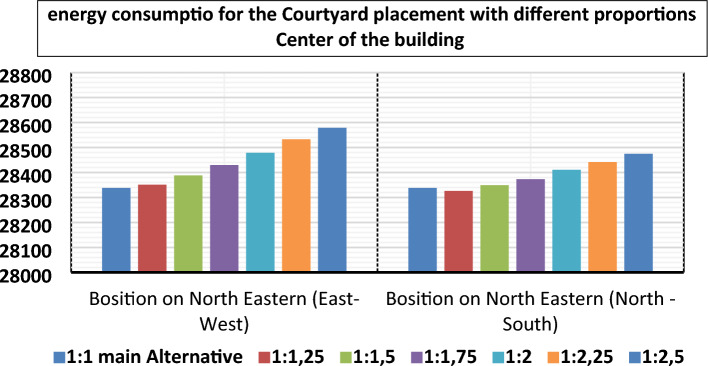
The total energy consumption of courtyard placements with different proportions in the center of the building.

**Figure 12:**
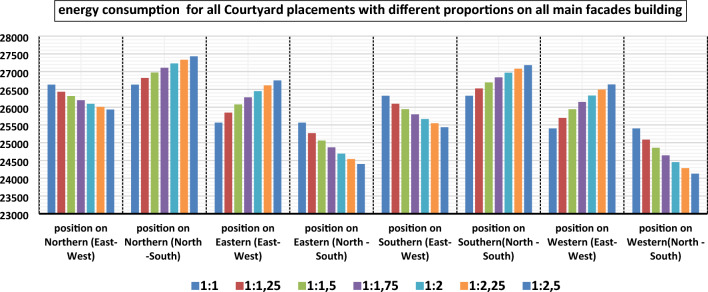
The total energy consumption of all building courtyard placements with different proportions on all main facades.

**Figure 13:**
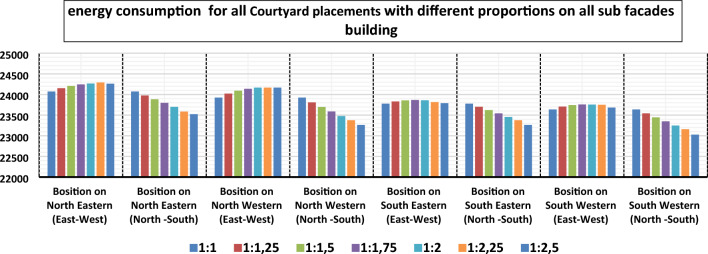
The total energy consumption of all building courtyard placements with different proportions on all other-facades.

The main results of the current work are presented in Tables 3 and 4 presents the simulation summary of the nine courtyard placements (the center of the building, the middle of the main facades Northern, eastern, southern, western, and on sub facades northeastern, northwestern, southeastern, southwestern) and different elongation (1:1, 1.25:1, 1.5:1–1.75:1, 2:1, 2.25:1, 2.5–1). Presenting energy consumption values for simulated cases of different courtyard placements for the selected housing model of Kharga, City in the New Valley. A comparison of the annual energy consumption for different courtyard placements is shown in Figs. 11, 12, and 13**.**

**Table 4:** The simulation summary of different courtyard positions.

Courtyard placement	Orientation of the longitudinal axis of the Courtyard	Length to width proportion	CO2 emissions (kg CO2)	Energy consumption (kwh)	The proportion of energy savings compared to the (main Alternative) %
Center of the building	**Main alternative**	**1:1**	**17,896**	**28,338**	**0%**
	North–South	1:1.25	17,889	28,326	0.04%
Northern position	East–West	1:2.5	16,451	25,934	8.48%
	North–South	1:1	16,872	26,634	6.01%
Eastern position	East–West	1:1	16,224	25,564	9.79%
	North–South	1:2.5	15,522	24,400	13.90%
Southern position	East–West	1:2.5	16,149	25,436	10.24%
	North–South	1:1	16,682	26,320	7.12%
Western position	East–West	1:1	16,125	25,401	10.36%
	North–South	1:2.5	15,356	24,128	14.86%
Northeastern position	East–West	1:1	15,328	24,072	15.05%
	North–South	1:2.5	14,995	23,523	17.00%
Northwestern position	East–West	1:1	15,239	23,926	15.57%
	North–South	1:2.5	14,837	23,263	17.91%
Southeastern position	East–West	1:1	15,150	23,779	16.09%
	North–South	1:2.5	14,837	23,263	17.91%
Southwestern position	East–West	1:1	15,064	23,637	16.57%
	**North**–**South**	**1:2.5**	**14,696**	**23,029**	**18.73%**

Bold values that give the best energy.

##### Regarding the courtyard placement in the center of the building, the results have suggested that:

The best length-to-width ratio of the Courtyard, which achieved a saving of 0.04%, is (1.25:1), the best orientation of the longitudinal axis of the Courtyard is the north–south direction (orientation angle is Zero), the less the length-to-width ratio is in the north–south direction, the less energy is consumed inside the building.

##### Regarding the courtyard placement at the northern and southern facades, the results have suggested that:

The best orientation of the longitudinal axis of the Courtyard is the east–west direction (orientation angle is Zero), the best length-to-width ratio of the Courtyard is (2.5:1), the highest rate of energy saving at the northern façade is 8.48%, while the highest rate of energy saving at the southern façade is 10.24%.

##### Regarding the courtyard placement at the eastern and western facades, the results have suggested that:

The best orientation of the longitudinal axis of the Courtyard is the north–south direction (orientation angle is Zero), the best length-to-width ratio of the Courtyard is (2.5:1), the highest rate of energy saving at the eastern façade is 13.90%, while the highest rate of energy saving at the western façade is 14.86%.

##### Regarding the courtyard placement at the (northeastern, northwestern, southeastern, and southwestern) facades, the results have suggested that:

The best orientation of the longitudinal axis of the Courtyard is the north–south direction (orientation angle is Zero), the best length-to-width ratio of the Courtyard is (2.5:1), the highest rates of energy saving in these placements are northeastern façade 17.00%, northwestern façade 17.91%, southeastern façade 17.91%, and southwestern façade 18.73%.

##### In addition to the analysis of the total energy consumption associated with the different design alternatives for the courtyard placements compared to the main Alternative

**Figure 14:**
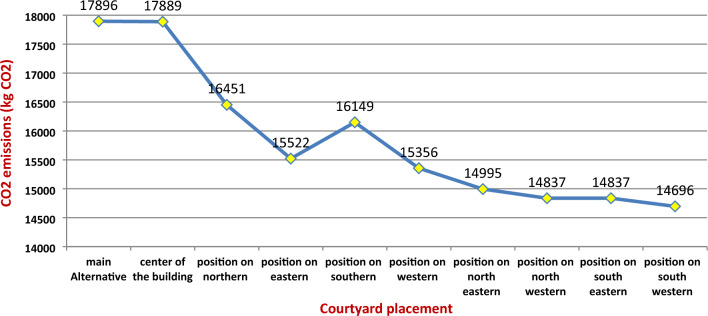
The predicted CO2 emissions reduction associated with the courtyard placements.

The CO2 emissions of cooling and heating loads have been calculated. Figure 14 summarizes the predicted CO2 emissions reduction associated with the courtyard placements. The basic Alternative achieved the highest percentage of CO2 emissions at a rate of 17,896 kg CO2/year, while the best location for the Courtyard had an annual CO2 emissions record of 14,696 kg CO2, decreasing 17.88%.

##### Table 4 the simulation summary for different courtyard positions the results in Table 4 suggest the following:

**Figure 15:**
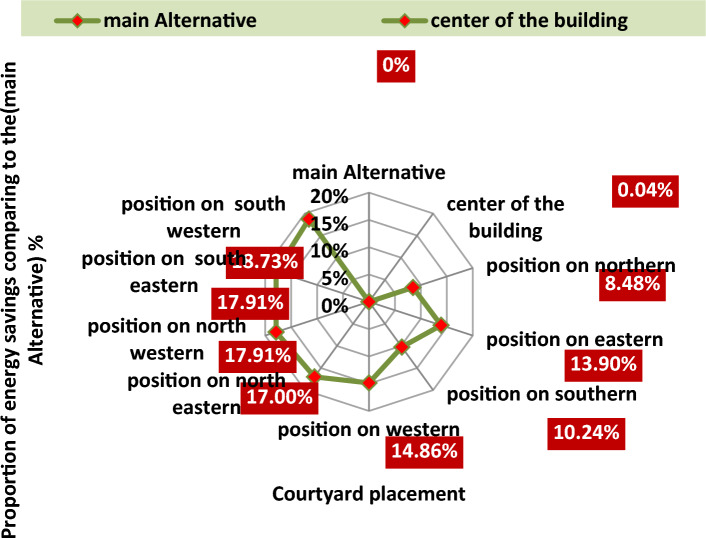
The Highest Energy Savings of Each Different Courtyard Placement.

Existence of a relationship between the courtyard placement and energy conservation, the best length-to-width ratio of the Courtyard that achieves the highest rate of energy consumption saving in case of the orientation of the longitudinal axis at the east–west direction and in case of the orientation of the longitudinal axis at the north–south direction for each of the courtyard placements, the best and worst Placement of the inner Courtyard in terms of energy consumption saving, as shown in Fig. 15.

## Conclusions

Through this study, we have concluded that the inner courtyards are one of the most important architectural elements consistent and appropriate to the desert hot regions. They could be used to attain thermal comfort inside architectural spaces and reduce the energy internally consumed in modern residential buildings through the most proper Placement and orientation of such a Courtyard. This study has paid attention to the simulation methods that have demonstrated the best designs of the Placement, orientation, and elongation of the Courtyard for residential buildings. The study has concluded with some important standards that could act as a methodological framework for designing modern residential buildings with inner courtyards that should be applied to such residential buildings in the hot dry climate.

Therefore, we recommend that the legislative authorities in Egypt enact laws that oblige architects to have an inner courtyard in any architectural design according to the design standards deduced from the research, ideal designs for the inner courtyard can lead to energy savings on a larger scale if they are applied in national housing projects and new cities, as residential buildings are considered the most energy-consuming types of buildings.

## The main outputs of this study could be briefed as follows:

1. The results suggest that the best Placement for the Courtyard among various design alternatives, which achieved the highest rate of saving of energy consumed inside the building, is the Placement of the Courtyard at the southwestern façade by a saving rate of 18.73%, compared to the main Alternative. The best length-to-width ratio is (2.5:1) if the longitudinal axis of the Courtyard is oriented in a north–south direction (orientation angle is Zero).
2. The results have demonstrated that the best orientation of the longitudinal axis at the following facades (eastern, western, northeastern, northwestern, southeastern, southwestern) is the north–south direction (orientation angle is Zero), and the greater the length-to-width ratio is at the north–South direction, the less energy is consumed inside the building.
3. In case of the orientation of the longitudinal axis in an east–west direction (orientation angle is Zero) for the following facades (eastern, western, northeastern, northwestern, southeastern, southwestern), the best length-to-width ratio of the Courtyard in this direction will be 1:1, i.e., the square shaped Courtyard is better than the rectangular shaped Courtyard in these placements.
4. The results have demonstrated that the best orientation of the longitudinal axis at the northern and southern facades is the east–west direction (orientation angle is Zero), and the greater the length-to-width ratio is in the east–west direction, the less energy is consumed inside the residence.
5. In case of the orientation of the longitudinal axis in a north–south direction (orientation angle is Zero) for the northern and southern facades, the best length-to-width ratio of the Courtyard in this direction will be 1:1, i.e., the square shaped Courtyard is better than the rectangular shaped Courtyard in these placements.
6. In comparison between the rates of energy saving in various design alternatives and the rate of energy saving in the main Alternative, the highest rate of energy saving is in the case of the courtyard placement at the southwestern façade by a saving rate of 18.73%, followed by the saving rate in the case of the courtyard placement at the southeastern and northwestern façades by a saving rate of 17.91%, in case of the orientation of the longitudinal axis of the Courtyard at the north–south direction (orientation angle is Zero), by a length-to-width ratio (2.5:1).

## Future research

The authors are highly recommended to study the following:

- The effect of courtyard geometry on energy consumption in residential buildings with vertical extension.
- The effect of courtyard geometry on energy consumption in residential buildings in wet areas.
- The effect of courtyard geometry on energy consumption in commercial and public buildings.
- The effect of the courtyard placement on the efficiency of the dwelling in contemporary urbanism.

## Data Availability

The datasets used and/or analyzed during the current study are available from the corresponding author on reasonable request.
